# Supplementary material for: A UMLS-based spell checker for natural language processing in vaccine safety
Source: BMC Med Inform Decis Mak. 2007 Feb 12;7:3. doi: 10.1186/1472-6947-7-3 (PMC1805499; doi:10.1186/1472-6947-7-3)
Supplement: Additional file 13 — RAP application directory. Sets up directory for RAP (RDF application for PHP) source code [file 1472-6947-7-3-S13.gz › rap/doc/phpdoc/__filesource/fsource_default__vocabularyRDFS.php.html]

xml version="1.0" encoding="iso-8859-1"?


File Source for RDFS.php


# Source for file RDFS.php

Documentation is available at RDFS.php

```
1. <?php
2. // ----------------------------------------------------------------------------------
3. // RDF Vocabulary Description Language 1.0: RDF Schema (RDFS) Vocabulary
4. // ----------------------------------------------------------------------------------
5. // Version                   : 0.9
6. // Authors                   : Daniel Westphal (dawe@gmx.de)
7. //
8. // Description               : Wrapper, defining resources for all terms of the
9. //                               RDF Schema (RDFS).
10. //                               For details about RDF see: http://www.w3.org/TR/rdf-schema/.
11. //                                Using the wrapper allows you to define all aspects of
12. //                             the vocabulary in one spot, simplifing implementation and
13. //                             maintainence. Working with the vocabulary, you should use
14. //                             these resources as shortcuts in your code.
15. //
16. // ----------------------------------------------------------------------------------
17. // History:
18. // 02-21-2003                 : Initial version
19. // ----------------------------------------------------------------------------------

21. // RDFS concepts

23. $RDFS_Resource = new Resource(RDF_SCHEMA_URI . 'Resource');
24. $RDFS_Literal = new Resource(RDF_SCHEMA_URI . 'Literal');
25. $RDFS_Class = new Resource(RDF_SCHEMA_URI . 'Class');
26. $RDFS_Datatype = new Resource(RDF_SCHEMA_URI . 'Datatype');
27. $RDFS_Container = new Resource(RDF_SCHEMA_URI . 'Container');
28. $RDFS_ContainerMembershipProperty = new Resource(RDF_SCHEMA_URI . 'ContainerMembershipProperty');
29. $RDFS_subClassOf = new Resource(RDF_SCHEMA_URI . 'subClassOf');
30. $RDFS_subPropertyOf = new Resource(RDF_SCHEMA_URI . 'subPropertyOf');
31. $RDFS_domain = new Resource(RDF_SCHEMA_URI . 'domain');
32. $RDFS_range = new Resource(RDF_SCHEMA_URI . 'range');
33. $RDFS_label = new Resource(RDF_SCHEMA_URI . 'label');
34. $RDFS_comment = new Resource(RDF_SCHEMA_URI . 'comment');
35. $RDFS_member = new Resource(RDF_SCHEMA_URI . 'member');
36. $RDFS_seeAlso = new Resource(RDF_SCHEMA_URI . 'seeAlso');
37. $RDFS_isDefinedBy = new Resource(RDF_SCHEMA_URI . 'isDefinedBy');


40. ?>
```

Documentation generated on Fri, 17 Dec 2004 16:17:07 +0100 by phpDocumentor 1.3.0RC3
